# Next generation sequencing in synovial sarcoma reveals novel gene mutations

**DOI:** 10.18632/oncotarget.5786

**Published:** 2015-09-22

**Authors:** Myrella Vlenterie, Melissa H.S. Hillebrandt-Roeffen, Uta E. Flucke, Patricia J.T.A. Groenen, Bastiaan B.J. Tops, Eveline J. Kamping, Rolph Pfundt, Diederik R.H. de Bruijn, Ad H.M. Geurts van Kessel, Han J.H.J.M. van Krieken, Winette T.A. van der Graaf, Yvonne M.H. Versleijen-Jonkers

**Affiliations:** ^1^ Department of Medical Oncology, Radboud University Medical Center, Nijmegen, The Netherlands; ^2^ Department of Pathology, Radboud University Medical Center, Nijmegen, The Netherlands; ^3^ Department of Human Genetics, Radboud University Medical Center, Nijmegen, The Netherlands; ^4^ The Institute of Cancer Research and The Royal Marsden NHS Foundation Trust, London, UK

**Keywords:** next generation sequencing, KRAS, CCND1, synovial sarcoma, chromosomal aberrations

## Abstract

Over 95% of all synovial sarcomas (SS) share a unique translocation, t(X;18), however, they show heterogeneous clinical behavior. We analyzed multiple SS to reveal additional genetic alterations besides the translocation. Twenty-six SS from 22 patients were sequenced for 409 cancer-related genes using the Comprehensive Cancer Panel (Life Technologies, USA) on an Ion Torrent platform. The detected variants were verified by Sanger sequencing and compared to matched normal DNAs. Copy number variation was assessed in six tumors using the Oncoscan array (Affymetrix, USA). In total, eight somatic mutations were detected in eight samples. These mutations have not been reported previously in SS. Two of these, in *KRAS* and *CCND1,* represent known oncogenic mutations in other malignancies. Additional mutations were detected in *RNF213, SEPT9, KDR, CSMD3, MLH1* and *ERBB4*. DNA alterations occurred more often in adult tumors. A distinctive loss of 6q was found in a metastatic lesion progressing under pazopanib, but not in the responding lesion. Our results emphasize t(X;18) as a single initiating event in SS and as the main oncogenic driver. Our results also show the occurrence of additional genetic events, mutations or chromosomal aberrations, occurring more frequently in SS with an onset in adults.

## INTRODUCTION

Synovial sarcoma (SS) accounts for approximately 8% of all soft tissue sarcomas. Synovial sarcomas occur at all ages and sites throughout the body, with a predilection for the extremities of young adults. Patients with a synovial sarcoma have a 5-year cancer-specific survival rate of 66%, with a remarkable better outcome for children as compared to adults [1]. Tumors can be aggressive, leading to early metastases and recurrences, or can be more indolent occurring as a long existing swelling that may recur years after the initial diagnosis [2]. Predicting tumor behavior has been attempted by relating survival to various tumor and patient characteristics. Several of these characteristics have been proven to be of negative prognostic value, including large tumor size, primary tumor location in non-extremities and older age at diagnosis. The mechanism(s) underlying the differences in tumor behavior, however, remain to be resolved [Vlenterie, et al. Abstract 022 presented at CTOS 2014]. The treatment of localized disease involves surgery often supplemented with (neo)adjuvant radiotherapy and, occasionally, with (neo)adjuvant chemotherapy or a combination of both. Metastatic disease is treated by palliative chemotherapy or by applying the angiogenesis inhibitor pazopanib, with limited survival benefit [3].

Genetic profiling is believed to be the way forward to explore tumor behavior and to discover new therapeutic targets. Currently, genetic screening is being implicated in standard clinical practice for several cancers, including melanomas, lung and colon cancers. Interestingly, similar mutations are shared by different cancer types, and within one cancer type different genetic subtypes can be found, explaining its biologic behavior and/or therapy response. In addition, differences in mutations have been observed between primary tumors and their metastases, thereby providing insight in tumor evolution and therapy resistance [4, 5]. Importantly, these insights have led to the development of new targeted therapies based on the use of monoclonal antibodies and tyrosine kinase inhibitors.

Sarcomas have also been subject to genetic screening, which has led to diagnostic implementation in several subtypes [6], including gene amplifications in well-differentiated and dedifferentiated liposarcomas [7] or distinct chromosomal translocations in, among others, myxoid liposarcomas [8]. Additionally, the discovery and treatment of the targetable alterations in hot spot regions in the *KIT* or *PDGFRA* genes in gastrointestinal stromal tumors (GIST) has significantly improved overall survival of these patients [9, 10].

A unique reciprocal translocation between chromosome X and 18 in over 95% of SS tumors was already reported in 1994, leading to fusions between one of the SSX genes (1, 2 or 4) and the SS18 (SYT) gene [11, 12]. This translocation is not found in any other human neoplasm. It has been shown that the SSX and SS18 (fusion) proteins participate in the SWI/SNF and Polycomb complexes, respectively, known to be involved in epigenetic gene (de)regulation [13, 14]. As knockdown of the fusion protein leads to cell death *in vitro* and *in vivo* and introduction of the translocation in mice forms histologically alike tumors [15] [16] [17], the translocation is believed to act as the central oncogenic driver in SS [18]. Next to its significance as a diagnostic marker, the clinical targeting of this translocation has so far remained elusive [19, 20]. Also the putative predictive value of SS translocation subtypes has stayed unclear [21, 22].

Besides the X;18 translocation, additional genomic alterations have been reported in SS. First, patients with Li-Fraumeni syndrome (loss of p53 function) or neurofibromatosis (altered function of *NF1* gene) have a higher risk for SS [23]. Secondly, Sanger sequencing of synovial sarcomas has revealed mutations in several cancer-related genes, including *TP53, TERT, CDH1, CTNBB1, APC, HRAS, PTEN, PI3KCA, EGFR, BCL9, SETD2, TRRAP* and *PDGFRA* (Table 1). The targeted sequencing of other cancer-related genes, including *KRAS* [24], *BRAF* [24, 25], *CDKN1A* [26], *KIT* [27] (abstract only), *JAK2, FOXL2, IDH1, AKT1* and *EZH2* [25] did not reveal any pathogenic mutations. Since the percentages of affected tumors differ widely in comparable studies its reproducibility may be questioned, and in most studies (10 of 15) the detected variations were not verified in the corresponding normal DNAs. Joseph et al. performed whole-exome sequencing of a small cohort of SS (*n* = 7), resulting in a relatively low mutation call [28]. Besides several mutations of unknown function, driver mutations in *TP53* and *SETD2* were found in one sample each. Thirdly, in addition to these nucleotide alterations, gross chromosomal aberrations have been detected by comparative genomic hybridization (CGH) [29-31], providing further insight into its genomic complexity next to the recurrent X;18 translocation. A recent array CGH (aCGH) and gene expression profiling study by Przybyl et al. in a subset of SS revealed up-regulation of the *AURKA* and *KIF18A* genes in aggressive untreated primary tumors and its corresponding metastases or local recurrences, compared to untreated primary tumors from patients who did not develop metastases/local recurrences [32]. Finally, the study of Lagarde et al. showed that there is a correlation between genomic complexity, based on the number and type of chromosomal aberrations, and metastasis-free survival. Their study also showed a relation to age at diagnosis, with a larger instability being more frequent in adults than children [33]. This observation could explain why children show better survival rates than adults.

**Table 1:** Previously reported non-synonymous mutations in synovial sarcoma

Gene	Examined part	Frequency	Sample size	Somatic	Reference
***TP53***	Exon 2-11	0	5	N/A	[47]
	Exon 5-9	6 **(12%)**	49	Not validated	[48]
	Unknown	1	1	Yes	[49]*
	Exon 5-8	2 **(6%)**	34	Not validated	[50]
***TERT***	C250T + C228T	1	25 + 5CL	Not validated	[51]
***EGFR***	Exon 18-21	1 **(8%)**	12	Not validated	[52]
	Exon 18-21	1 **(50%)**	2	Yes	[53]
***CDH1***	Exon 4-9	12 **(24%)**	49	Yes	[54]
	Exon 4-9	5 **(12.5%)**	40	Not validated	[55]
	Unknown	1 **(6%)**	16	Not validated	[56]
***CTNNB1***	Exon 3	4 **(8%)**	49	Not validated	[57]
	Exon 3	0	15	N/A	[58]
	Unknown	2 **(12.5%)**	16	Not validated	[56]
	Exon 3	1 **(20%)**	5CL	Not validated	[37]
***APC***	Exon 15	4 **(8%)**	49	Yes	[59]
	Unknown	0	16	N/A	[56]
	Coding region	0	5CL	N/A	[37]
***HRAS***	Codon 12 and 13	3 **(6%)**	49	Not validated	[48]
***PTEN***	Exon 5-9	2 **(7%)**	30	Not validated	[24]
	Unknown	7 **(14%)**	49	Unknown	[60]*
***PI3KCA***	Exon 9 and 20	2 **(7%)**	30	Not validated	[24]
	Exon 1, 9, 20	0	23	N/A	[61]
	Exon 9, 20	0	8	N/A	[25]
***PDGFRA***	Exon 12 & 18	1 **(8%)**	12	Not validated	[27]*
	Exon 12-16 & 18-21	0	25 + 2CL	N/A	[38]
Exome	mutation in SETD2, TP53, TRRAP, BCL9 and other mutations in non cancer related genes	1 **(14%)**	7	Yes	[28]

* abstract only; CL=cell lines

Here we used next generation sequencing in a relatively large SS cohort to assess the occurrence of genomic alterations, including mutations and gross chromosomal changes.

## RESULTS

We included patients of all ages (range 11-78 years; including 8 children (< 18years) and 19 adults (≥18years)) and both sexes (female: 15, male: 22). Patients were diagnosed between 1990 and 2014. Follow-up data was available for 32 (86%) patients. For the screening of somatic mutations by means of next generation sequencing, we included 26 tumors from 22 patients (cohort 1) of whom sufficient tissue with matched normal tissue was available in the local tissue bank from our hospital pathology database. We included tumors with both histology and translocation subtypes (Table 2). The tumors encompassed 18 primary tumors, 6 metastatic tumors and 2 recurrences. Seventy-seven % (*n* = 20) of the tumor samples were from chemotherapy naïve patients, three tumor samples were from patients treated with neo-adjuvant chemotherapy, one couple consisting of two metastatic lesions were derived from one patient treated with pazopanib, and one recurrence was from a localization previously treated with adjuvant radiotherapy. Of four patients paired lesions were available: three patients with a metastasis and the corresponding primary tumor, and one patient, as mentioned above, with two metastases that responded differently to pazopanib.

**Table 2:** Patient characteristics

	Cohort 1 (n=22)	Cohort 2 (n=15)	Patients with a mutation
**Age**			
**• Children (<18 years)**	6 (27.3%)	2 (13.3%)	1 (12.5%)
**• Adult (≥18 years)**	16 (72.7%)	13 (86.7%)	7 (24.1%)
			*(p = NS)*
**Sex**			
**• Male**	16 (72.7%)	6 (40%)	6 (27.3%)
**• Female**	6 (27.3%)	9 (60%)	2 (13.3%)
			(*p = NS)*
**Tumor localization**			
**• Extremity**	12 (54.5%)	9 (40%)	4 (19.0%)
**• Non-extremity**	9 (40.9%)	6 (60%)	4 (26.7%)
**• Unknown**	1 (4.5%)	0	0
			*(p = NS)*
**Histology**			
**• Monophasic**	11 (50%)	10 (66.7%)	4 (19.0%)
**• Biphasic**	8 (36.4%)	3 (20%)	3 (27.3%)
**• Unknown**	3 (13.6%)	2 (13.3%)	1 (20.0%)
			*(p = NS)*
**Translocation**			
**• SSX1**	14 (63.6%)	11 (73.3%)	6 (24.0%)
**• SSX2**	8 (36.4%)	4 (26.7%)	2 (16.7%)
			*(p = NS)*
**Follow up**			
**• 5 year overall survival**	62%	67%	29.2%
			*(p = 0.026)*

NS = not significant (*p* > 0.05)

**Figure 1:**
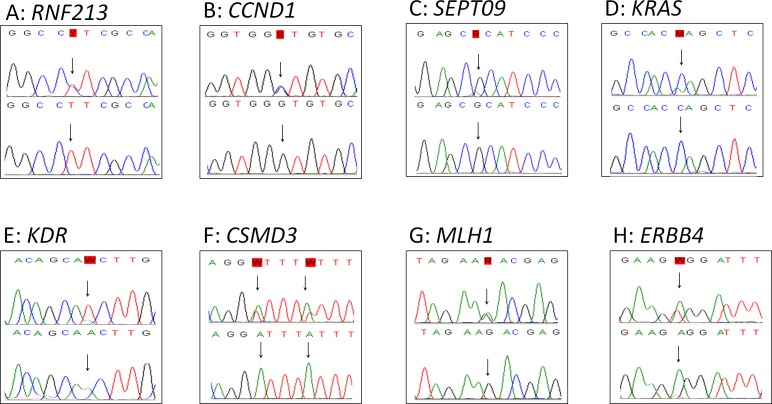
Sanger verification Figure 1 shows the 8 verified mutations by Sanger sequencing in tumor tissue (top) with the corresponding normal tissue (bottom).

**Figure 2:**
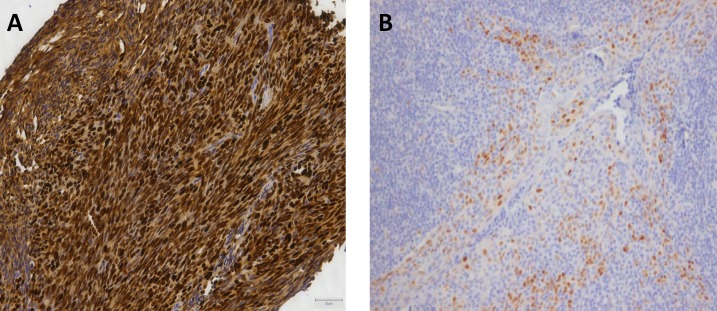
Immunohistochemistry of cyclin D1 Figure 2A shows the abundant overexpression of cyclin D1 by immunohistochemical staining. 2B is the positive control (tonsil). Photos are made with 20x enlargement.

The 26 tumors were sequenced using the Comprehensive Cancer Panel, containing 409 cancer-related genes including all previous found mutated genes in SS, except for *TERT* and *CDKN1A*. In total 77,995 variants were called in the 26 tumors (range 755 - 5713). 96 single nucleotide variants remained after filtering. These remaining variants were verified by Sanger sequencing. Of these, 57 variants (59%) could be confirmed. All were compared to matched normal DNA. In total, 49 of the 57 variants were also detected in normal DNA and were, thus, considered to be polymorphisms. Eight variants were not found in the normal tissues and are therefore assigned as somatic mutations (Table 3). These mutations were identified in the genes: *KRAS*, *CCND1*, *RNF213*, *SEPT9*, *KDR (VEGFR2)*, *CSMD3*, *MLH1* and *ERBB4 (HER4)* (Figure 1). Seven of these mutations were found in primary tumor samples derived from therapy naïve patients. The *KRAS* mutation was found in a tumor sample from a patient who was treated with neo-adjuvant chemotherapy. The mutations in the oncogenes *KRAS* and *CCND1* genes are well-established oncogenic mutations in other cancer types. The sample harboring the *CCND1* mutation was further evaluated by immunohistochemistry, showing abundant over-expression of the protein (Figure 2). The sample harboring the *MLH1* mutation was also evaluated by immunohistochemistry for MLH1, MSH2, MSH6 or PMS2 expression in accordance to the effect of MLH1 in Lynch [34]. However, no lack of expression of any of these proteins was found (data not shown) in the tumor sample with the *MLH1* mutation.

**Table 3:** Sanger verified mutations

Nr	Gene		Chr	Exon	HG19 notation	mRNA	Amino acid	Type
**1A**	*RNF213*	Ring finger protein 213	17	29	78319549	c.7414T>C	p.F2472L	Missense
**1B**	*CCND1*	Cyclin D1	11	5	69466021	c.859C>G	p.P287A	Missense
**1C**	*SEPT09*	Septin-9	17	5	75483596	c.1004G>A	p.R335H	Missense
**1D**	*KRAS*	V-Ki-ras2 Kirsten rat sarcoma viral oncogene homolog	12	2	25398285	c.34C>A	p.G12C	Missense
**1E**	*KDR* (a.k.a. *VEGFR2*)	Kinase insert domain receptor	4	18	55963862	c.2581T>A	p.T861S	Missense
**1F**	*CSMD3*	CUB and Sushi multiple domains 3	8	2	114327017	c.184TAAAT>AAAAA	p.IF62FF	Missense
**1G**	*MLH1*	MutL homolog 1	3	12	37067195	c.1106C>T	p.S369F	Missense
**1H**	*ERBB4* (a.k.a. *HER4*)	Receptor tyrosine-protein kinase erbB-4	2	25	212285269	c.3032T>A	p.E2V	Missense

No additional mutations were found in the metastatic lesions compared to the primary tumor. Since all mutations were found only once, we extended our cohort with a second cohort (Cohort 2, total *n* = 15), one primary tumor sample was derived from a patient who was treated with neo-adjuvant chemotherapy, the rest were primary tumor samples from therapy naïve patients, to test whether the identified mutations might occur recurrently. In addition, we extended the sequencing with coding exons 1-5 of the *CCND1* gene and coding exons 2-5 of the *KRAS* gene, as the mutations that we found in these genes are proven pathogenic and, therefore, these two genes were considered to be of particular interest. However, no additional mutations were found in cohort 2 which means that after analysis of 41 tumors with these platforms, at maximum one mutation was found in each tumor, and no similar mutations were observed.

**Figure 3:**
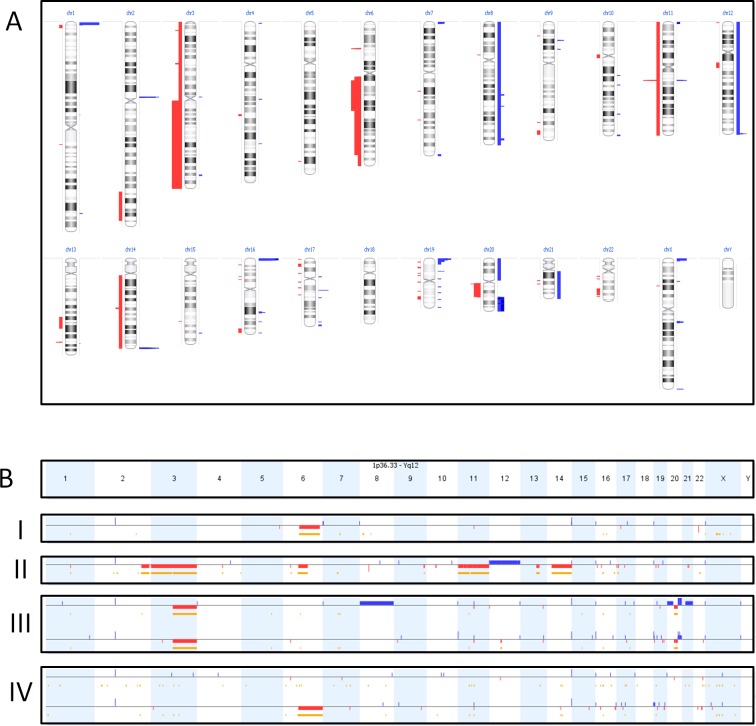
Oncoscan results Figure 3A shows an overview of the number of copy-number-variations in 6 synovial sarcomas with aggregated gains (blue) and losses (red) of the different cases. The width of the bars indicates the number of cases with the gain or loss. Figure 3B shows the copy number variations per sample (one per row). Gains and losses of the different chromosomes are represented by respectively blue and red lines, under the different chromosomes (depicted in columns). The length of these lines indicates the size of the gain or loss. The yellow/orange lines indicate loss of heterozygosity. BI and BII are two individual lesions showing a partial loss of chromosome 6q. BIII is a primary lesion (top line) with its corresponding metastasis (bottom line), showing a partial overlap of chromosomal aberrations but also differences. BIV are two metastases from the same patient. It shows a new deletion of chromosome 6q in the progressive metastasis under pazopanib treatment (bottom) compared to the metastasis responding to pazopanib (top).

In addition to the above mutation screen, we also used the primary dataset of cohort 1 to search for the presence of copy number variations (CNVs). To this end, we compared the aligned number of reads per gene generated by NGS in tumor tissue with the aligned number of reads per gene in healthy tissue. By doing so, chromosomal aberrations, like partial loss of chromosome 3 or gain of chromosome 8, were detected in approximately half of the SS. We confirmed these results in 6 samples with genome wide CNV analysis using the Oncoscan FFPE assay from Affimetrix (Figure 3A). Interestingly, differences in copy number alterations were seen in 2 of the 4 paired tumor lesions (Figure 3B) with additional deletions, duplications and loss of earlier duplications between the primary tumor and its corresponding metastasis. A specific loss of 6q was found in the metastatic lesion which progressed under pazopanib treatment in contrast to the responding metastatic lesion. An additional (partial) deletion of chromosome 6q was found in 2 other tumors of cohort 1. Another recurrent finding was loss of heterozygosity (LOH) in 5 of the 6 tumor samples at 3q13.33. Overall, tumors with chromosomal aberrations were more frequently seen in adults (34.5%) compared to children (12.5%), however this was not significant (*p* = 0.07).

## DISCUSSION

Synovial sarcoma is a rare sarcoma subtype, which is characterized by a recurring X;18 translocation. Tumors show a heterogeneous clinical behavior. An in-depth genetic characterization may lead to an explanation of the clinical differences in tumor behavior and, ultimately, the identification of new therapeutic targets.

Using a next generation sequencing platform, we detected pathogenic mutations that have so far not been reported in synovial sarcoma. In contrast to earlier reports, all mutations were unique in nature, and no recurrent mutations were found. Also, no mutations were found in previously reported mutated genes in SS (Table 1).

Although each mutated gene may be involved in tumorigenesis, only two mutations that we identified in the *KRAS* and *CCDN1* genes are presently known to be functionally important for driving cancer. Cyclin D1, encoded by the *CCND1* gene, is a cell cycle regulator. It can associate with the cyclin-dependent kinases (CDKs) CDK4 and CDK6 to phosphorylate the retinoblastoma protein (RB) during the G1 phase of the cell cycle. Phosphorylation of cyclin D1 at threonine at codon 286 is required for its ubiquitination, nuclear export and degradation. Mutations at codon 287, by which the proline changes to threonine or serine, have been reported in endometrial carcinomas [35]. These mutations result in nuclear accumulation of the active Cyclin D1/CDK complex, which is refractory to rapid degradation via the 26S proteasome. In our cohort, we identified a c.859C > G (p.P287A) mutation and, concomitantly, we observed a cyclin D1 accumulation by immunohistochemistry. The protein encoded by the *KRAS* gene is involved in recruiting and activating proteins necessary for the propagation of growth factor and other receptor signaling, such as c-RAF and PI3-kinase. The single nucleotide substitution c.34C > A that we found represents an activating mutation known to result in oncogenesis in several adenocarcinomas. Non small cell lung cancer cell lines with this mutant had activated phosphatidylinositol 3-kinase (PI3K) and mitogen-activated protein/extracellular signal-regulated kinase kinase (MEK) signaling [36]. Both mutations are involved in different pathways known to be activated in SS, i.e. the WNT - β-catenin pathway which targets CCND1[37], and KRAS targeting the PI3K pathway [38]. The effect of the other mutations is not clear and therefore they could be passenger-mutations. As all eight mutations are found in genes involved in different pathways, including regulation of EGFR degradation (*SEPT9*) [39] or the EGFR-pathway (ERBB4) [40], angiogenesis (*KDR* [41], *RNF213* [42]), proliferation (*CSMD3* [43]) and mismatch repair (*MLH1* [34]) pathways, no uniform suitable therapeutic target has emerged. The 5-year overall survival was significantly worse in patients whose tumor harbored an additional mutation, which should be further investigated in a larger cohort. Mutations were more often found in adult tumors compared to tumors with an onset in patient younger than 18 years (Table 2).

Besides mutations, structural chromosomal aberrations have also been reported in SS. In our cohort, approximately half of the samples showed chromosomal aberrations. Some tumors had multiple alterations whereas others showed only a few or none. Also both large, including whole chromosomes, and small alterations were found. Similarly as Lagarde et al. reported, we found more stable genomes in children compared to adults [33]. Probably due to our small cohort this was not significant in our study. It is unknown if the amount of chromosomal aberrations is related to the aggressiveness of the tumor or a cause of the aggressive behavior, as genomic instability itself is related to aging and related to cancer [44]. Also, Chakiba et al. evaluated a possible association between genomic instability and response to chemotherapy, but no relation was found [45]. The deletion of 6q that we found may be an exception that typically raises interest in resistance mechanisms to pazopanib. As the working mechanism of pazopanib in sarcomas is still not unraveled, resistance may occur in various pathways, including anti-angiogenic pathways. Partial 6q loss has been reported before in SS [29-31, 46], but so far no clinical correlation was found between 6q loss (or any other chromosomal aberration) and the clinical behavior of SS.

Our study underlines the diversity in SS genomes beyond the well-known X;18 translocation. It emphasizes the challenge in finding new druggable targets in this disease and encourages a personalized medicine approach because of the overlapping mutations with other cancer types. As was shown by the sets of primary tumor and metastases, tumor evolution is unlikely to be explained by additional mutations although our sample size was small, but change in chromosomal alterations can be found. This study also warrants further investigation of a putative correlation between chromosomal aberrations (i.e. deletion of 6q) and resistance to pazopanib. From this study we conclude that not only mutations or copy number changes may underlie the immense complexity of human cancers, including SS, and, based on our and previous results, also further epigenetic research might be a way to explore the genetic nature of SS.

## MATERIALS AND METHODS

### Patients and tissue samples

Tumor samples were obtained from the archives of the Department of Pathology at the Radboud University Medical Center, Nijmegen (1990-2013). In total, 36 frozen and 5 formalin-fixed, paraffin embedded (FFPE) tumor tissue samples, representing 37 patients, were included. In all patients the specific t(X;18) translocation was identified by reverse transcriptase polymerase chain reaction (RT-PCR). Patient follow-up was retrieved from clinical records. All research was performed in consultation and agreement with the medical ethical committee.

### Mutation analysis

Genomic DNA was extracted by incubating the frozen/FFPE tissue samples in 5% Chelex-100 in lysis buffer and proteinase K twice overnight. All samples were examined by a pathologist to evaluate the neoplastic cell load: all tumor cases contained more than 70% neoplastic cells. The control samples did not contain neoplastic cells. Extracted DNA samples were quantified using the Qubit (Invitrogen) and quality was checked by size ladder PCR before library preparation. Libraries were generated using Life Technologies Ion AmpliSeq™ Comprehensive Cancer Panel according to the manufacturer's recommendations. This panel consists of approximately 16 000 primer pairs covering 409 genes with known cancer associations. 10ng of genomic DNA from each sample was used to prepare barcoded libraries using IonXpress barcoded adapters (Life Technologies). Libraries were combined to a final concentration of 3ng/ml using the Ion Library Quantification Kit (Life Technologies, USA), and emulsion PCR was performed using the Ion Torrent OneTouchTM 2 System. Samples were sequenced on the Ion Torrent semi-conductor sequencer (Life Technologies, USA) using Ion 316 or 318 chips. Sequencing reads were aligned to the 409 genes based on the Human Genome version 19 using Sequence Pilot v4.2.0 (JSI medical systems GmbH). Also read depth and uniformity of coverage across individual amplicons were assessed.

In data analysis the cut-off was set at mutations found in ≥20% of the reads. Only non-synonymous and non-sense variations in coding regions were included. Mutations were filtered for known single nucleotide polymorphisms and variations found earlier in our own research database. All mutations left after filtering were confirmed by Sanger sequencing with specifically designed primer sets (Supplemental Data 1), and if confirmed, the presence or absence of this specific mutation was verified in normal non-neoplastic tissue from the corresponding patient (extracted from FFPE normal tissue). PCR reactions were performed using the AmpliTaq Gold 360 Master Mix (Life Technologies, USA) with 1 μl DNA and the following program: 95°C (10 min); 95°C (30 sec), 58/60°C (30 sec), 72°C (1 min), 38 cycles; and 72°C for 7 min. PCR products were analyzed by agarose gel electrophoresis. Subsequently, samples were submitted to DNA sequencing using the BigDye Terminator reaction mix, and samples were analyzed on the 3730 Sequence Analyzer (Applied Biosystems). All validation was done in duplicate, including the DNA extraction process.

We extended our cohort with cohort 2 (*n* = 15) and analyzed these by Sanger-sequencing for the mutations that were identified in the first cohort. We also included all coding exons of *KRAS* and *CCND1* (primers are listed in Supplemental Data 1).

### Oncoscan

DNA was extracted from 3 FFPE and 3 frozen tissues and purified with ethanol precipitation to a concentration of 12 ng/ul. The samples were processed with the OncoScan™ FFPE Assay, a whole-genome copy number assay, according to the manufacturers' protocol of the OncoScan^tm^ FFPE Assay Kit Protocol by Affymetrix. The data was analyzed with Nexus Copy Number 7.5.2, standard edition, BioDiscovery, Inc. 2014.

### Immunohistochemistry

4 μm sections of FFPE tissue were pretreated in a PreTreatment module (Lab Vision) in either sodium citrate buffer (pH6.7) for 30 min at 100°C (CCND1) or in ethylenediaminetetraacetic acid (EDTA) buffer (pH9) for 10 min at 96°C (MLH1, MSH2, MSH6, PMS2). After blocking of endogenous peroxidase with 3% hydrogen peroxide in methanol, sections were incubated for 1h at room temperature (RT) with the primary antibody against Cyclin D1 (ILM 30442, clone SP4; 1:40 dilution; Immunologic), MLH1 (551092, clone G168-15; 1:40 dilution; BD Pharmingen), MSH2 (NA26, clone GB12; 1:40 dilution; Calbiochem), MSH6 (ab92471, clone EPR3945; 1:500 dilution; Abcam) or PMS2 (556415, clone A16-4; 1:100 dilution; BD Pharmingen). Next, sections were incubated with PowerVision poly-HRP-anti-Ms/Rb/Rt (Immunologic) for 30 min at RT and visualized using bright 3,3′-diaminobenzidine (DAB). Counterstaining was performed with haematoxylin. Immunostaining was evaluated by a pathologist.

## SUPPLEMENTARY MATERIAL TABLE


