# Supplementary material for: Next generation sequencing in synovial sarcoma reveals novel gene mutations
Source: Oncotarget. 2015 Sep 22;6(33):34680–90. doi: 10.18632/oncotarget.5786 (PMC4741482; doi:10.18632/oncotarget.5786)
Supplement: Supplementary file 1 [file oncotarget-06-34680-s001.pdf]

Next generation sequencing in synovial sarcoma reveals novel gene mutations

Supplementary Material

Supplemental table: Primer sequences. *KRAS c.34C>A primers contain a M13 sequence (underlined). The annealing temperature of this PCR is 58°C, all other PCRs had an annealing temperature of 60°C.

Primer	Sequence 5'-3'	PCR product size (bp)
***ERBB4* 3032T>A FW**	CGTATGAAGCTTCCCAGTCC	111
***ERBB4* 3032T>A RV**	GAAAGCCTGAGGGACCAAGT	
***RNF213* 7414T>C FW**	CGGAGGAACAACTGCAGACA	128
***RNF213* 7414T>C RV**	TAGCTTCCGTTGTGTTGGCT	
***CSMD3* 184TAAAT>AAAAA FW**	gtgcccagctcttaaactgc	178
***CSMD3* 184TAAAT>AAAAA RV**	GTCCTTTTAAAGTTCCACCACA	
***KDR* 2581T>A FW**	ttgactctagGTAAGCCTCTTGG	104
***KDR* 2581T>A RV**	TTGACTGCTACTGTCCTGCAA	
***SEPT09* 1004G>A FW**	GGCTTGGGTAAATCCACCTT	119
***SEPT09* 1004G>A RV**	TGCGTGATGGACTTGATCTC	
***MLH1* 1106C>T FW**	TGGGGAGATGGTTAAATCCA	132
***MLH1* 1106C>T RV**	AGAGGCTGCAGAAATGCATC	
***CCND1* 859C>G FW**	AGAACATGGACCCCAAGG	182
***CCND1* 859C>G RV**	GGAGAGGAGGGACTGTCAGG	
KRAS* 34C>A FW	TGTAAAACGACGGCCAGTAGGCCTGCTGAAAATGACTG	115
KRAS* 34C>A RV	CAGGAAACAGCTATGACCTGGATCATATTCGTCCACAAAA	
***KRAS* exon 2 FW**	cgtctgcagtcaactggaat	339
***KRAS* exon 2 RV**	agaatggtcctgcaccagtaa	
***KRAS* exon 3 FW**	cagactgtgtttctcccttctca	285
***KRAS* exon 3 RV**	tgcatggcattagcaaagac	
***KRAS* exon 4 FW**	tgacaaaagttgtggacaggtt	390
***KRAS* exon 4 RV**	aagaagcaatgccctctcaa	
***KRAS* exon 5 FW**	cttcttgcacatggctttcc	274
***KRAS* exon 5 RV**	gtggttgccaccttgttacc	
***CCND1* exon 1 FW**	AGCTGCCCAGGAAGAGC	270
***CCND1* exon 1 RV**	gcaacaagttgcagggaagt	
***CCND1* exon 2 FW**	ctgccaagcgcgatg	416
***CCND1* exon 2 RV**	tcggaggagcagatatgtca	
***CCND1* exon 3 FW**	ccttgagtccccagcattc	441
***CCND1* exon 3 RV**	ggacatcttcccagacagca	
***CCND1* exon 4 FW**	acagcctccttccctctctc	203

CCND1 **exon 4 RV**	ctgggacatcaccctcactt	
CCND1 **exon 5 FW**	acctctccccaccctctct	275
CCND1 **exon 5 RV**	GGAGAGGAGGGACTGTCAGG	
